# *In vitro* culture with gemcitabine augments death receptor and NKG2D ligand expression on tumour cells

**DOI:** 10.1038/s41598-018-38190-2

**Published:** 2019-02-07

**Authors:** Andrew M. Gravett, Angus G. Dalgleish, John Copier

**Affiliations:** Oncology Group, Institute for Infection and Immunity, St. George’s, University of London, London, UK

## Abstract

Much effort has been made to try to understand the relationship between chemotherapeutic treatment of cancer and the immune system. Whereas much of that focus has been on the direct effect of chemotherapy drugs on immune cells and the release of antigens and danger signals by malignant cells killed by chemotherapy, the effect of chemotherapy on cells surviving treatment has often been overlooked. In the present study, tumour cell lines: A549 (lung), HCT116 (colon) and MCF-7 (breast), were treated with various concentrations of the chemotherapeutic drugs cyclophosphamide, gemcitabine (GEM) and oxaliplatin (OXP) for 24 hours *in vitro*. In line with other reports, GEM and OXP upregulated expression of the death receptor CD95 (fas) on live cells even at sub-cytotoxic concentrations. Further investigation revealed that the increase in CD95 in response to GEM sensitised the cells to fas ligand treatment, was associated with increased phosphorylation of stress activated protein kinase/c-Jun N-terminal kinase and that other death receptors and activatory immune receptors were co-ordinately upregulated with CD95 in certain cell lines. The upregulation of death receptors and NKG2D ligands together on cells after chemotherapy suggest that although the cells have survived preliminary treatment with chemotherapy they may now be more susceptible to immune cell-mediated challenge. This re-enforces the idea that chemotherapy-immunotherapy combinations may be useful clinically and has implications for the make-up and scheduling of such treatments.

## Introduction

One way that chemotherapies can sensitise tumour cells to immune-mediated apoptosis is by modifying the interaction between CD95 (FAS/APO-1) and its cognate ligand, FasL (CD178, CD95). Ligation of CD95 with FasL is an important factor in immune-mediated clearance of cancer as this underpins the apoptosis of diseased cells by cytotoxic effector cells, such as natural killer cells (NKs) and αβ or γδ T-cells. Activation of these immune effectors causes upregulation of FasL on the plasma membrane, increasing their cytolytic potential and when FasL on an effector cell ligates with CD95 on the surface of a target cell, such as a tumour cell, it instigates formation of a death-induced signalling complex and initiation of the caspase proteolytic cascade. This ultimately leads to the apoptosis of the target cell.

As the CD95 death receptor plays such a critical role in apoptosis, and especially in immune-mediated apoptosis, its expression is significant in the development and treatment of cancers. Tumour cells have developed a variety of ways to either avoid CD95-induced death or turn it to their advantage. Tumours can escape immune surveillance through suppression of CD95 signalling, release of soluble decoy receptors to bind to and inactivate FasL on immune cells^1^ and may even induce apoptosis in immune effectors directly by expressing FasL themselves. A common method for tumours to avoid CD95-mediated cell death is by reducing surface expression of the CD95 protein^2^, this eliminates apoptotic signals by preventing interaction with FasL but can be reversed with agents such as the nucleoside analogue 5-azacytidine if the downregulation of CD95 is caused by methylation of DNA^3^. Despite CD95 being implicated in the maintenance and aggressiveness of some cancers^4,5^, it may also be seen as a positive prognostic factor, as many studies have highlighted^6–9^.

Initiating apoptosis in tumour cells through methods involving the augmentation of CD95 signalling is a treatment avenue that has been explored, mostly unsuccessfully, for a number of years^10^ but a large swathe of evidence exists suggesting that chemotherapies sensitise tumours to CD95/FasL killing^11,12^ and CD95 has been shown to be upregulated upon cells after treatment with a number of chemotherapeutics including: doxorubicin, cisplatin, mitomycin C, 5-FU, dacarbazine and gemcitabine^13,14^. This phenomenon is observed in numerous cancers and is thought to be dependent of functional wild-type p53, either increasing translation of the *FAS* gene or translocation of the protein to the plasma membrane. Other molecules associated with immune-sensitivity, such as TRAIL receptors (TRAILRs) and NKG2D ligands have also been reportedly induced by chemotherapies in various cancer types^15,16^.

The work presented here aims to show whether chemotherapies, including the antimetabolite nucleoside analogue gemcitabine (GEM) which is primarily used in pancreatic, non-small cell lung, breast and ovarian cancers and has been used experimentally in colorectal cancers, can increase expression of CD95 on the surface of a panel of tumour cell lines and whether any increase is functional in terms of induced-cell death. Moreover, in-line with recent reports additional signs of immune sensitivity will be explored in terms of expression of death receptors and immune effector ligands.

## Materials and Methods

### Cell Culture

The human cancer cell lines; A549 (lung), HCT116 (colon) and MCF-7 (breast) (Public Health England, Porton Down, UK), were grown in complete medium, DMEM (Sigma-Aldrich, Dorset, UK), supplemented with 10% foetal bovine serum (FBS) (Invitrogen, Paisley, UK), 2 mM and 1% penicillin/streptomycin (Sigma). For all experiments cells were seeded at 1 × 10^5^ cells/ml and allowed to attach overnight before addition of drugs or other reagents for 24 hours.

### Drugs, Inhibitors and CD95 cross-linking reagents

GEM, oxaliplatin (OXP) and cyclophosphamide (CPM) (Sigma) were reconstituted in phosphate buffered saline (PBS) (Sigma). ERK signalling was inhibited with U0126 (New England Biolabs, Hitchin, UK) while SP600125 (Sigma) was used to block the JNK pathway. For experiments involving ligation of CD95, his-tagged CD95L was used at 50 ng/ml with a cross-linking polyhistidine monoclonal antibody (both R & D Biosystems, Abingdon, UK) at 3 μg/ml. Ligation of CD95 was blocked using an antibody antagonistic to CD95 (Prospec, East Brunswick, USA).

### Flow Cytometric Analysis

Cells were stained with fluorochrome-conjugated antibodies specific for CD95 (Biolegend, London, UK); ULBP2/5/6 (R & D) and TRAILR 1 and 2 (Biolegend). MICA/B was stained using an unconjugated primary antibody and anti-species secondary antibody (both Biolegend). Cells were washed prior to resuspending in Cellfix (Becton Dickinson (BD), Oxford, UK). Acquisition of data was performed within 24 hours using an LSRII flow cytometer (BD Biosciences) by gating on live cells and measuring median fluorescence intensity (MFI).

### MTT Assay

The methylthiazoletetrazolium (MTT) assay was used to measure cell number. Briefly, 0.4 mg/ml MTT (Sigma) was added to cell cultures and plates incubated for 60 minutes. After this time, medium was aspirated off, 200 μl DMSO added to each well and plates agitated gently for before measuring optical density at 540 nm using a microplate reader (Dynex-MRX II, Dynex Technologies Ltd. West Sussex, UK)).

### Illumina microarrays

RNA was isolated from HCT116 cells using the Qiagen (Manchester, UK) mini-kit protocol following manufacturer’s instructions. Microarrays were performed by Dr Jayne Dennis at the St. George’s, University of London Biomics Centre. Biotinylated cRNA was generated from 100 ng total RNA using the Illumina TotalPrep RNA Amplification Kit (Applied Biosystems, Warrington, UK) according to manufacturer’s instructions. Equal amounts (750 ng) of cRNA were hybridised to the Illumina human HT12-v3 arrays for 18 hours and subsequently processed according to manufacturer’s instructions before scanning on an Illumina BeadArray Reader. The image data were processed using default values in GenomeStudio v2009.1 with imputation of missing data, before loading onto GeneSpring v9.0 for data normalisation and filtering.

### Cignal Reporter Assay

The Cignal Finder™ RTK 10-Pathway Reporter Array (Qiagen) was used to assess activation of various signalling pathways in HCT116 cells. The manufacturer’s suggested protocol was followed with some modifications. Briefly, 50 μl of Opti-MEM^®^ medium was added to each well of the array plate to resuspend the signalling-pathway-related transcription-factor-responsive reporter and control constructs. Then, 0.5 μl lipofectamine^®^ LTX™ in 50 μl Opti-MEM^®^ medium was added to the plate before incubating for 20 minutes at room temperature. HCT116 tumour cell suspension was then added at 3.5 × 10^4^ cells/ml. The plate was incubated overnight, before culturing for a further 24 hours with or without the addition of GEM. The transfected cells were cultured with GEM for zero (untreated), one, four or 24 hours. Pathway-specific transcription factor activity in response to GEM was determined using the Dual-Luciferase^®^ Reporter Assay System (Promega, Southampton, UK) following manufacturer’s instructions. Luminescent activity from each sample was quantified with a Promega GloMax^®^ Multi + Detection Reader.

## Results

### Chemotherapy induces expression of CD95 in tumour cell lines

Our previous studies showed an increase in expression of MHC class I on selected tumour cell lines in response to relatively low concentrations of GEM. Also observed were alterations in other components of the antigen processing machinery^17^ suggesting that a coordinated alteration of immunophenotype is occurring in GEM-treated cells. Here we sought to confirm previous data suggesting that DNA-damaging chemotherapies, including GEM, have been shown to increase CD95 on tumour cells^18^. This was tested with HCT116, A549 and MCF-7 cell lines *in vitro*.

**Figure 1:**
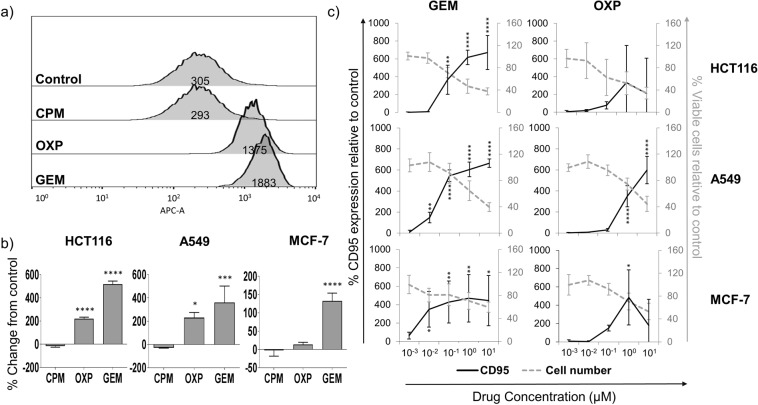
Flow cytometry was used to measure levels of CD95 on the surface of tumour cells after 24 hour culture with equi-active (in terms of cytotoxicity) concentrations of chemotherapeutic drugs. (**a**) Representative flow cytometry histograms showing change in MFI values in response to chemotherapy drugs for the HCT116 cell line. (**b**) Histograms showing change in CD95 at the cell surface for each cell line. Data were normalised to untreated controls, which were assigned a value of 0% change. (**c**) Dose response showing change in CD95 on the plasma membrane and cell number when cell lines were cultured with 1 nM – 10 µM GEM or OXP. For (**b**) and (**c**), mean and standard deviation is plotted from at least three separate experiments and CD95 values are significantly different (****p < 0.0001, ***p < 0.001, **p < 0.01, *p < 0.05) to controls by one-way ANOVA with Dunnett’s multiple comparisons test.

Flow cytometry was used to measure CD95 levels on the surface of live tumour cells after 24 hour culture with drugs at previously reported equi-active cytotoxic concentrations: CPM – 100 μM for all cell lines, OXP – 1 μM for A459 and HCT116 cells and 0.6 μM for MCF-7 cells and GEM – 1 μM for A549, 0.6 μM for HCT116 cells and 0.3 μM for MCF-7 cells^19^. CD95 was significantly increased in all three cell lines in the presence of GEM (Fig. 1b). The increase in CD95 was most prominent in the HCT116 cell line, where levels were upregulated by a mean of 518% compared to untreated controls. Culturing cells with CPM resulted in no change from the basal level of CD95 at IC_25._ For all cell lines, CD95 was upregulated in a dose-dependent manner (Fig. 1c) in response to GEM or OXP, however, increases in CD95 were achieved at lower concentrations of GEM in A549 and MCF-7 cells; both cell lines approximately doubling (100% increase) CD95 on the plasma membrane in the presence of 10 nM GEM. HCT116 and A549 cells had a higher maximum relative increase in CD95 than MCF-7 cells, around 7-fold in the former cell lines versus only 4-fold in MCF-7 cells. CD95 was also increased after culture with OXP, especially in the HCT116 and A549 cell lines, although all of the cell lines were less sensitive to CD95 upregulation by OXP compared to GEM and a statistically significant increase was not observed for the HCT116 cell line. Trypan blue cell counts showed that increased CD95 was associated with a reduction in the number of viable cells, linking the upregulation of CD95 to growth inhibitory effects of the drugs (Fig. 1c).

### GEM-mediated CD95 upregulation is reduced in the presence of JNK inhibitor

**Figure 2:**
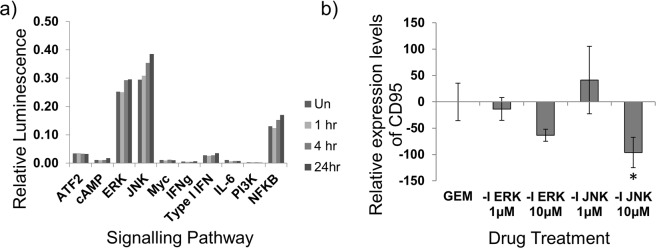
Analysis of cell signalling activity after culturing tumour cells with GEM. (**a**) The activation of various signalling pathways in HCT116 tumour cells was assessed after exposure to 500 nM GEM for one, four or 24 hours. The experiment was completed in triplicate for: zero, one and four hours, or duplicate for 24 hours and mean values are shown. Un = Untreated control (0 hours), 1 hr, 4 hr and 24 hr indicate the length of time the cells were exposed to GEM. (**b**) HCT116 cells were cultured with 100 nM GEM, with or without addition of inhibitors (-I) for the ERK and JNK pathways. After 24 hours, the effect on CD95 was measured by flow cytometry using MFI values. Data represent change in the amount of CD95 at the cell surface in response to GEM compared to matched inhibitor only controls and are shown relative to the no inhibitor control. Mean and standard deviations from five separate experiments are shown. *Denotes value significantly different from control by one-way ANOVA (p < 0.05).

The intracellular signalling pathways that underlie GEM-mediated upregulation of CD95 were investigated by assessing the phosphorylation status of signalling proteins in HCT116 cells cultured with GEM. Figure 2a shows that culturing tumour cells with GEM increased signalling through pathways involving the kinases ERK and JNK as shown by increases in the activity of the transcription factors Elk-1/SRF and AP-1, respectively. The maximum increase in signalling for most pathways was achieved at 24 hours, with only the JNK pathway showing any activation at the earliest time-point of one hour. In order to determine whether signalling through these pathways played a role in CD95 upregulation, cell lines were cultured with GEM and either an inhibitor of the ERK pathway, U0126, or the JNK pathway, SP600125. Figure 2b shows that blocking signalling through the JNK pathway significantly inhibited GEM-mediated CD95 upregulation. HCT116 cells were cultured +/− GEM and +/− U0126 or SP600125 and any change in the level of CD95 from controls was assessed by flow cytometry. Data points shown here represent the change from controls (untreated or in the presence of U0126 or SP600125 alone) to GEM-treated (+/− U0126 or SP600125) and are expressed relative to the change in CD95 from untreated to GEM-treated cells. CD95 upregulation was reduced by more than a third by blocking JNK signalling (*p < 0.05). Inhibiting the ERK pathway did not significantly alter GEM-mediated upregulation of CD95.

### The CD95 upregulation induced by GEM is functionally active and ligation of it causes a marked decrease in cell number

**Figure 3:**
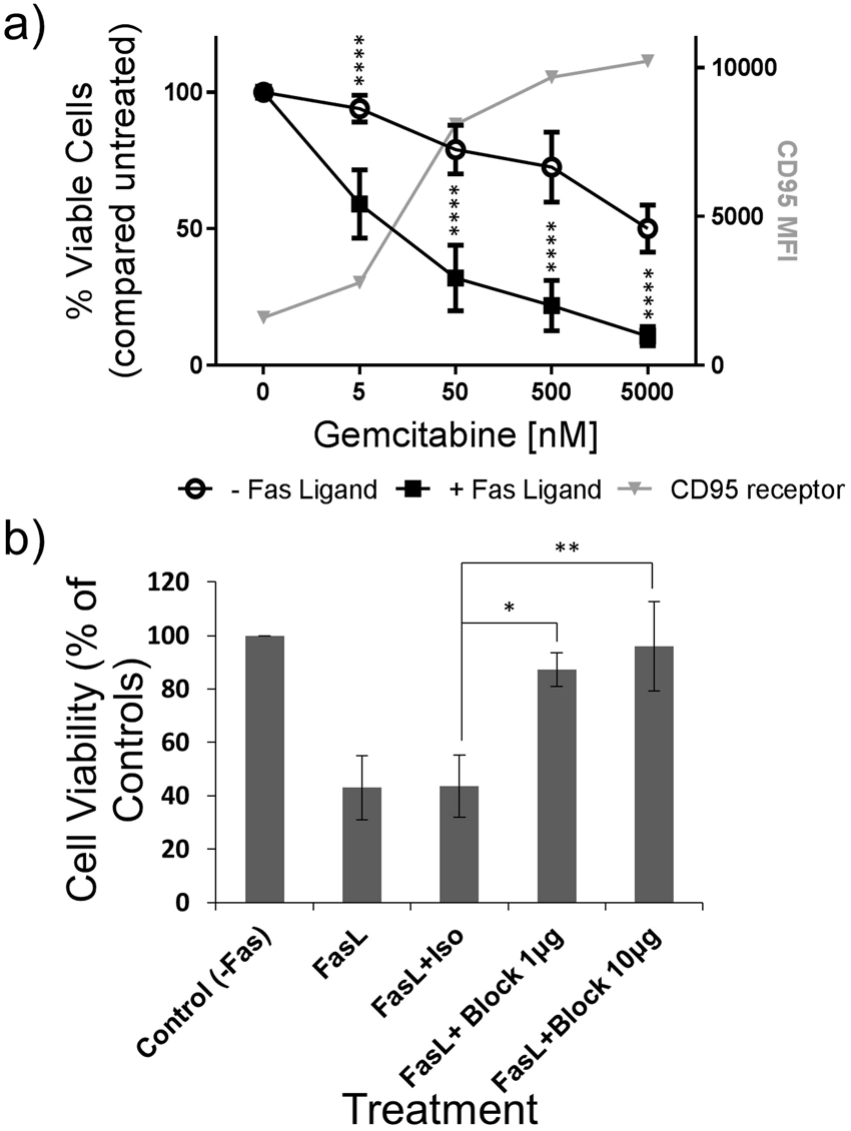
Combining GEM and CD95 ligation in HCT116 cells. (**a**) Viable cell number was measured by the MTT assay in the presence of various concentrations of GEM for 24 hours with or without the addition of FasL. Data were normalised to 0 nM GEM controls. Graphs represent the means and standard deviations of three separate experiments and values significantly different in the presence of FasL by two-way ANOVA with Sidak’s test multiple comparison test are indicated (****p < 0.0001). Also shown is the effect of GEM on CD95 levels. (**b**) Cells were treated with a single concentration of both GEM and CD95L and cell number measured as before. The interaction between CD95 and FasL and, hence, CD95-mediated cell death was blocked using an antagonistic antibody for CD95. An irrelevant isotype matched antibody was used as a control. Graphs represent the means and standard deviations of three separate experiments and values significantly different to control by one-way ANOVA with Dunnett’s multiple comparison test are shown.

To determine whether GEM-mediated increases in CD95 were functionally relevant, HCT116 cells were cultured with GEM in the presence or absence of soluble his-tagged FasL and a cross-linking polyhistidine antibody. The number of viable cells was then measured using the MTT assay. Addition of FasL to untreated cells resulted in a very small decrease in cell number but where GEM caused increases in CD95, combining with FasL greatly reduced the number of viable cells. This decrease in cell number was likely mediated through the CD95 apoptotic pathway due to increased interaction between CD95 on tumour cells and FasL in the medium. In cultures with 5 nM GEM there was a relatively modest 57.7% increase in CD95 compared to controls but the number of viable cells was markedly reduced by the addition of FasL to the culture (Fig. 3a). At this concentration of GEM, 94.1% of the number control cells were still viable with no FasL present, but this was reduced to only 59.1% when FasL was included in the culture. At 50 nM GEM, where expression of CD95 was greatly increased, the difference in viable cell number between cell cultures in the absence or presence of FasL was even greater, changing from 79.0% to 32.1% of controls, respectively.

Figure 3b shows that by blocking CD95-FasL interaction the decrease in cell number caused by combining GEM with FasL was negated. A concentration of 10 nM GEM was used in all tests, including the control, which in this instance means without FasL. The reduction in cell number measured with GEM alone was negligible but when FasL was added to the culture a large decrease was observed, down to 40% of the GEM alone control. However, addition of a CD95 blocking antibody restored cell number to similar levels as the GEM-alone control. This suggests that CD95-mediated cell death, through ligation of FasL with its receptor, is responsible for the reduced cell number observed in GEM-FasL combinations and that GEM-induced CD95 is functional as a death receptor.

### Other markers of immunogenicity

DNA damaging chemotherapies are known to induce stress-related innate immune receptors other than CD95. Therefore, next we assessed whether additional molecules involved in the sensitivity of tumour cells to immune cell killing were altered by culturing with GEM.

Firstly, microarray of GEM-treated HCT116 cells (Table 1) suggested that increases in surface CD95 protein were associated with increased transcription of the *FAS* gene. *FAS* mRNA increased 2.3-fold in response to GEM. Additionally, microarray showed increased mRNA of the death receptor genes *TRAILR1* and 2, and immune cell activatory NKG2D ligands, such as MICB and ULBP2 upon culture with GEM. Expression of *TRAILR1 (TNFRSF10A*) mRNA was increased 1.4-fold and *TRAILR2 (TNFRSF10B*) 1.8-fold. While of the TRAIL decoy receptors *TNFRSF10C* was unchanged and *TNFRSF10D* increased 1.5-fold, though these values were only just above the detection limit of the assay. Additionally, transcription of key NKG2D ligands *MICB* (1.6 fold) and *ULBP2* (2.0 fold) was increased by culturing cells with GEM as measured by microarray.

**Table 1:** Microarray data showing how the mRNA levels of various molecules associated with immune recognition and killing are altered by culturing HCT116 tumour cells with 100 nM GEM for four hours.

Gene	Untreated	Treated	Fold change
**Death Receptors**			
*FAS*	137.6	320.9	2.3
*TNFRSF10A*	560.5	765.2	1.4
*TNFRSF10B*	997.6	1750.0	1.8
**NKG2D Ligands**			
*MICA*	218.1	234.3	1.1
*MICB*	529.5	833.8	1.6
*ULBP1*	218.7	240.7	1.1
*ULBP2*	195.1	397.9	2.0

**Figure 4:**
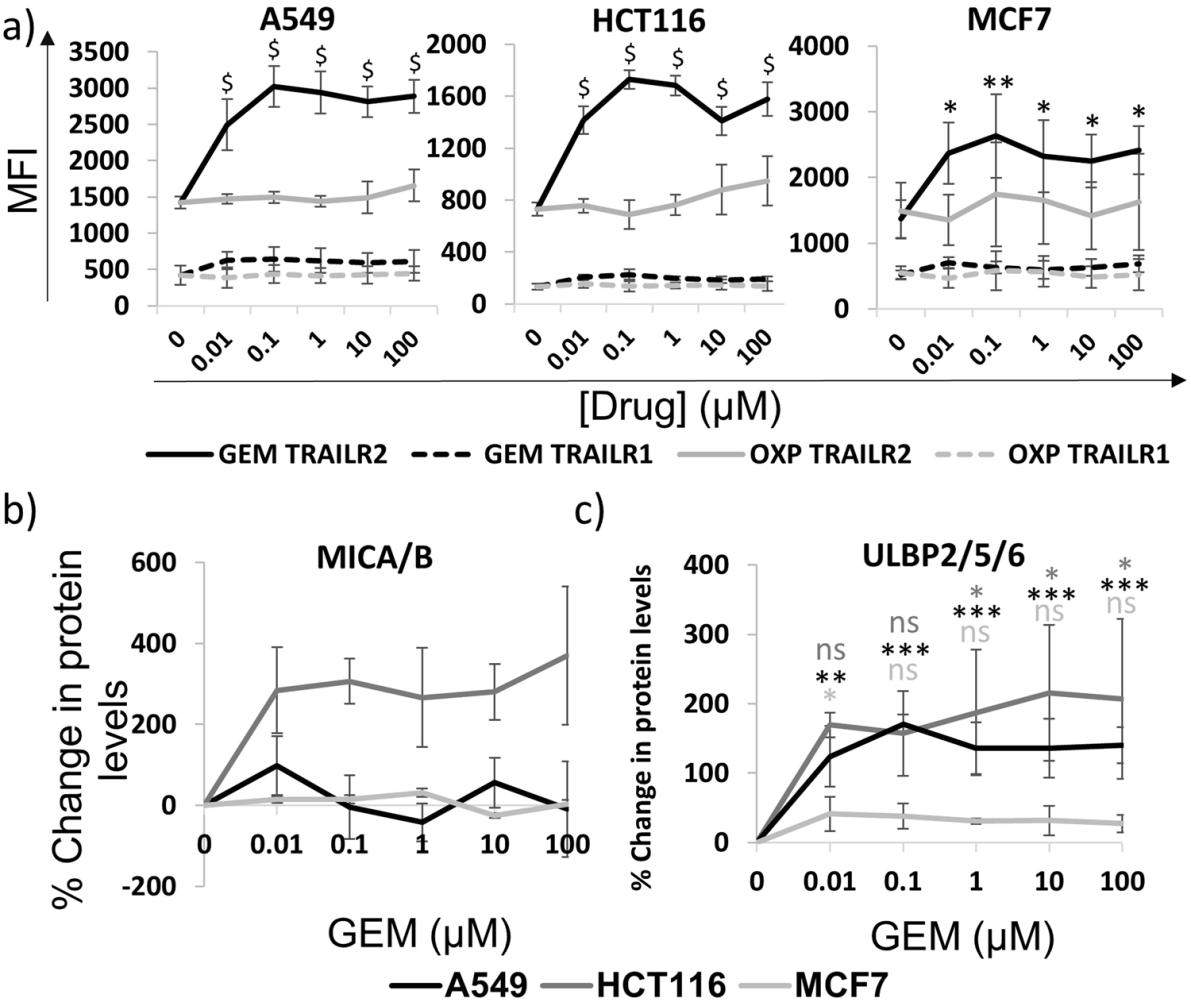
Molecules that are associated with immune recognition and killing are altered by culturing tumour cell lines with GEM. (**a**) Graph showing how levels of TRAILR1 (dashed) and TRAILR2 (solid) on the plasma membrane changes in the presence of GEM (black) and OXP (grey). Data represent MFI values from flow cytometry analysis of three separate experiments. Values significant from matched untreated control values (0 μM) by two-way ANOVA with Dunnett’s test for multiple comparisons are indicated (*p < 0.05, **p < 0.01, ^$^p < 0.0001). Expression of MICA/B (**b**) and ULBP2/5/6 (**c**) was measured using flow cytometry after 24 hour culture in the presence of GEM. Data points represent MFI values expressed relative to untreated controls and represent the mean and standard deviation of three separate experiments. Significance from untreated controls by one-way ANOVA with Dunnett’s test for multiple comparisons is shown (ns = non-significant, *p < 0.05, **p < 0.01, ***p < 0.001) and colour-coded to indicate cell line: HCT116 (dark grey, top), A549 (black, middle) and MCF-7 (light grey, bottom).

Furthermore, Fig. 4a shows that TRAILR2 protein was also increased at the surface of cells in response to GEM treatment. Expression of TRAILR1 was undetectable above isotype control in all cell lines and conditions tested. In contrast to its effects on CD95, OXP had no effect on the expression of the TRAIL death receptors TRAILR1 or TRAILR2. The observed increase in *MICB* mRNA expression in response to GEM was associated with increased MICA/B protein on HCT116 cells, though this did not reach significance. MICA/B expression was not upregulated upon A549 and MCF-7 cells (Fig. 4b). Adding to the complexity of stress-molecule-related cell line responses to GEM, ULBP2/5/6 was strongly increased on the surface of HCT116 and A549 cells but not MCF-7 cells (Fig. 4c). The increased levels of death receptors and NKG2D ligands may render the tumour cells more sensitive to lysis by αβ T-cells, γδ T-cells and NK cells by increasing the chance that an interaction between an immune effector cell and a tumour cell will result in the death of the tumour cell.

## Discussion

In concordance with other reports, culture with GEM led to increased levels of CD95 on the surface of tumour cells. This has been previously observed *in vitro*^20^ and *in vivo* using aerosolised GEM^21^. However, the upregulation of CD95 in the present study was more pronounced and achieved at lower concentrations than in earlier *in vitro* reports. This is also the first time that such a response has been shown in HCT116, A549 and MCF-7 cell lines. The increases in CD95 were functional, as culture of GEM-treated cells with soluble CD95L led to an augmented reduction in cell number compared to GEM alone. GEM-mediated increases in CD95 were associated with alterations in the phosphorylation status of the stress-induced kinase, JNK.

Apoptosis of colorectal cancer cells in response to direct culture with drugs such as GEM and OXP is reported to result in an increase in CD95 on the surface of the cells^20,22^. GEM and OXP were also shown to increase expression of CD95 on the colorectal, breast and lung tumour cell lines tested in the present study, however, this was not necessarily associated with apoptosis as these changes in CD95 occurred at concentrations were cell number was unaffected. It has been known for some time that DNA-damaging cancer drugs have the ability to increase innate immune receptor expression on tumour cells; so the upregulation of CD95 in these cell lines was no surprise given that they express functional p53, which is thought necessary for activating the CD95 gene upon chemotherapy-induced DNA damage^23^. The increases in surface CD95 protein levels observed in the present study were dose dependant, but, importantly, even small increases in CD95 in response to GEM seemed to enable efficient killing of tumour cells in the presence of FasL.

Previous studies have reported that expression of CD95 can be decreased in lung carcinoma compared to non-neoplastic tissue^24^. Therefore, whether the increases observed here are a correction to “normal” or an increase above basal levels is unknown but the margin of CD95 upregulation and associated cell death with relatively low concentrations of FasL suggests the latter. In line with previous reports^25^, after augmenting expression of CD95 with chemotherapy, agonising the CD95 receptor with FasL reduced cell number. This suggested that the increase in CD95 protein observed at the surface of tumour cells was functionally able to transmit apoptotic signals and this occurred even with the modest CD95 augmentation triggered by low concentrations of GEM. Some reports have suggested that the combination of GEM and FasL actually induces a necroptotic form of cell death rather than classical apoptosis but this is yet to be tested^26^.

The sensitisation of tumour cells to FasL-mediated cytotoxicity demonstrated in the present study may partly explain the cooperation between GEM treatment and immunotherapy. Especially when GEM treatment, which raises CD95 on tumour cells, is combined with activated immune effector cells (which should express FasL), such as LAK cells^27,28^. It may be that the clinical efficacy of GEM and other drugs could be greatly enhanced by supplementing with a FasL signal. This could be achieved through inducing FasL expression on immune cells, using an activating immune therapy, or by using fusion proteins of FasL, such as CD40-FasL or CTLA-4-FasL. These fusion proteins have had some success in killing malignant cell lines alone *in vitro*^29^, but this was dependent on the tumour cells constitutively expressing high levels of CD95. The work presented here suggests that inducing higher level of CD95 through culture with GEM may endow a whole new raft of tumour cells with capacity for cell death mediated through the CD95/FasL axis. The same may be true for apoptosis induced through TRAIL.

Previous studies have implicated JNK phosphorylation as a consequence of signalling through the CD95 receptor. Here, it is shown that JNK phosphorylation is also important in the upregulation of CD95. It may be that treatment with GEM activates the JNK pathway (which is often activated in response to cellular stress) which induces CD95 upregulation. Any subsequent signalling through CD95 could create a positive feedback loop, with signalling through JNK increasing expression of CD95 to amplify the cell death (or proliferative) signal still further.

In addition to the effects on CD95, expression of other protein markers associated with the susceptibility of cells to immune-mediated cytotoxicity were altered in response to chemotherapeutic drugs. TRAILR2 was increased at the surface of tumour cells by GEM treatment. Raising the amount of this death receptor may represent another avenue that can be exploited by the immune system to clear tumour cells. A previous report has shown the sensitisation of HCT116 tumour cells to TRAIL-mediated clearance using chemotherapy^30^. Although chemotherapeutic or genotoxic stresses have been implicated in the upregulation of all of the molecules identified as being upregulated by GEM, this investigation may be the first time that a single agent has been shown to increase CD95, TRAILR2, MICA/B and ULBP2/5/6 on tumour cells. A similar study indicating that treatment with 5-FU or doxorubicin could sensitise colon cancer stem cells to Vγ9Vδ2 T-cell cytotoxicity did look for these markers at the mRNA level but found that only TRAILR2 was significantly upregulated^31^. Whether molecules other than TRAILR2 were upregulated at the cell surface is unknown. Where, in the Todaro study 5-FU and doxorubicin increased TRAILR2 but failed to increase CD95, in the present investigation OXP was shown to increase CD95 but not TRAILR2, suggesting that the modulation of immune sensitivity markers is not a general effect of chemotherapeutics *per se* but differing mechanisms of action exist dependent on the type of chemotherapy and cell-type used.

In addition to augmenting death receptor expression, immune-mediated cytotoxicity of tumour cells may be enhanced by increased recognition of tumours through upregulation of proteins such as ligands of NKG2D. GEM-treated tumour cells were found to express increased amounts of the NKG2D ligands ULBP2 and MICA/B at their surface and microarray analysis suggested this was due to increased transcription induced by GEM treatment. ULBPs and MICA/B are NKG2D ligands involved in enhancing killing by cytotoxic lymphocytes such as αβ and γδ T-cells and NK cells, so, their upregulation on tumour cells is relevant to the immune response to tumour. NKG2D ligand upregulation is linked to DNA damage and cellular stress, such as caused by chemotherapy^32,33^. Indeed, similarly to the present study a recent report states that low dose GEM can induce MICA/B expression on some pancreatic cancer cell lines^34^. A further observation from the study by Miyashita *et al*. was that soluble MICA/B was released by tumour cells in response to treatment with GEM and that this enhances innate immune cell function. It is possible that the increased gene expression of *MICB* observed in the present study may also lead to the release of soluble MICB from the cell, although this is as yet untested. Higher expression of NKG2D ligands is linked to a better prognosis for cancer patients^35,36^.

In contrast to the upregulation of death receptors and NKG2D ligands, cellular stress can induce tumour cells to produce immunosuppressive factors such as IL-10, TGF-β and PD-L1 and these can negatively affect the activation of- and killing by- immune cells. It is the balance of these activatory and inhibitory signals that determines whether the cell is deleted by the immune system. Whether GEM also causes the type of stress that stimulates tumour cells to heighten their immunosuppressive environment has not been fully investigated but initial experiments have shown that there is no detectable GEM-mediated increase in surface levels of PD-L1 or release of IL-10 in the cell lines tested here and that mRNA of the aforementioned genes is also not increased (data not shown).

The upregulation of CD95 and other markers on the surface of tumour cells may indicate that the cells are “searching” for a signal to die. One that is provided in the present study by the addition of FasL to the GEM cultures. The upregulation of *FAS, TRAILR1/2, MICB* and *ULBP2* genes together in response to GEM suggests there may be a coordinated biological strategy by which cells containing DNA damage become sensitised towards immune-cell killing. GEM seems to prime tumour cells for cell death through CD95 or TRAIL, but the biological relevance of increasing the expression of these death receptors on tumour cells will depend on an additional signal provided by the presence of FasL or TRAIL expressing CTLs or NK cells in the tumour milieu, that can induce apoptosis via cognate ligation. Taken together with our previous work which showed that GEM induced immunoproteasomes and altered the peptides that were displayed on HLA-molecules^17^, it suggests that tumour cells treated with GEM are primed for immune-mediated cytotoxicity in a number of ways. These effects may be associated with the DNA damage response in response to GEM.

The present study is not the first time that chemotherapy, or indeed GEM, has been shown to sensitise tumour cells to immune-mediated death. But the upregulation of a number of surface proteins key to cell cytotoxicity through the actions of relatively low concentrations of a single chemotherapeutic agent implies a coordinated response to this type of genotoxic stress and permits the activation state of the immune system to ultimately determine the fate of a damaged cell. This is something which may be beneficial in tumour or infected cells where normal damage-evaluation and apoptotic pathways are hijacked. The ability of GEM to induce expression of these molecules together may be one of the reasons for the success of this chemotherapeutic and have particular relevance to the interactions of this drug with immunotherapies.

## Data Availability

There are no restrictions to the availability of materials and data. Accession number for microarray data: GSE122985.

## References

[1] Ashkenazi A (2002). Targeting death and decoy receptors of the tumour-necrosis factor superfamily. Nat Rev Cancer.

[2] Boldrini L (2002). Identification of Fas (APO-1/CD95) and p53 gene mutations in non-small cell lung cancer. Int J Oncol.

[3] Wu J, Wood GS (2011). Reduction of Fas/CD95 promoter methylation, upregulation of Fas protein, and enhancement of sensitivity to apoptosis in cutaneous T-cell lymphoma. Arch Dermatol.

[4] Hadji A (2014). Death induced by CD95 or CD95 ligand elimination. Cell Rep.

[5] Peter ME (2015). The role of CD95 and CD95 ligand in cancer. Cell Death Differ.

[6] de Carvalho-Neto PB (2013). FAS/FASL expression profile as a prognostic marker in squamous cell carcinoma of the oral cavity. PLoS One.

[7] Sträter J (2005). Impaired CD95 expression predisposes for recurrence in curatively resected colon carcinoma: clinical evidence for immunoselection and CD95L mediated control of minimal residual disease. Gut.

[8] Baryshnikov AYu (1999). CD95 (FAS/APO-1) antigen is a new prognostic marker of blast cells of acute lymphoblastic leukaemia patients. Adv Exp Med Biol.

[9] Mottolese M (2000). Prognostic relevance of altered Fas (CD95)-system in human breast cancer. Int J Cancer.

[10] Villa-Morales M, Fernández-Piqueras J (2012). Targeting the Fas/FasL signaling pathway in cancer therapy. Expert Opin Ther Targets.

[11] Costa-Pereira AP, Cotter TG (1999). Camptothecin sensitizes androgen-independent prostate cancer cells to anti-Fas-induced apoptosis. Br J Cancer.

[12] Park MA (2010). Vorinostat and sorafenib increase CD95 activation in gastrointestinal tumor cells through a Ca(2+)−de novo ceramide-PP2A-reactive oxygen species-dependent signaling pathway. Cancer Res.

[13] Yang S, Haluska FG (2004). Treatment of melanoma with 5-fluorouracil or dacarbazine *in vitro* sensitizes cells to antigen-specific CTL lysis through perforin/granzyme- and Fas-mediated pathways. J Immunol.

[14] Micheau O, Solary E, Hammann A, Martin F, Dimanche-Boitrel MT (1997). Sensitization of cancer cells treated with cytotoxic drugs to fas-mediated cytotoxicity. J Natl Cancer Inst.

[15] Elrod HA, Sun SY (2008). Modulation of death receptors by cancer therapeutic agents. Cancer Biol Ther.

[16] Fionda C, Soriani A, Zingoni A, Santoni A, Cippitelli M (2015). NKG2D and DNAM-1 Ligands: Molecular Targets for NK Cell-Mediated Immunotherapeutic Intervention in Multiple Myeloma. Biomed Res Int.

[17] Gravett, A. M., Trautwein, N., Stevanović, S., Dalgleish, A. G. & Copier, J. Gemcitabine alters the proteasome composition and immunopeptidome of tumour cells. *OncoImmunology*, e1438107 (2018). 10.1080/2162402X.2018.1438107 PMC5990974 29930882

[18] Siena L (2014). Gemcitabine sensitizes lung cancer cells to Fas/FasL system-mediated killing. Immunology.

[19] Liu WM, Fowler DW, Smith P, Dalgleish AG (2010). Pre-treatment with chemotherapy can enhance the antigenicity and immunogenicity of tumours by promoting adaptive immune responses. Br J Cancer.

[20] Christgen M, Schniewind B, Jueschke A, Ungefroren H, Kalthoff H (2005). Gemcitabine-mediated apoptosis is associated with increased CD95 surface expression but is not inhibited by DN-FADD in Colo357 pancreatic cancer cells. Cancer Lett.

[21] Gordon N, Kleinerman ES (2010). Aerosol therapy for the treatment of osteosarcoma lung metastases: targeting the Fas/FasL pathway and rationale for the use of gemcitabine. J Aerosol Med Pulm Drug Deliv.

[22] Almendro V (2009). The role of MMP7 and its cross-talk with the FAS/FASL system during the acquisition of chemoresistance to oxaliplatin. PLoS One.

[23] Müller M (1998). p53 activates the CD95 (APO-1/Fas) gene in response to DNA damage by anticancer drugs. J Exp Med.

[24] Viard-Leveugle I, Veyrenc S, French LE, Brambilla C, Brambilla E (2003). Frequent loss of Fas expression and function in human lung tumours with overexpression of FasL in small cell lung carcinoma. J Pathol.

[25] Symes JC, Kurin M, Fleshner NE, Medin JA (2008). Fas-mediated killing of primary prostate cancer cells is increased by mitoxantrone and docetaxel. Mol Cancer Ther.

[26] Pietkiewicz, S., Eils, R., Krammer, P. H., Giese, N. & Lavrik, I. N. Combinatorial treatment of CD95L and gemcitabine in pancreatic Cancer cells induces apoptotic and RIP1-mediated necroptotic cell death network. *Exp Cell Res* (2015). 10.1016/j.yexcr.2015.10.005 26453936

[27] Hirooka Y (2009). A combination therapy of gemcitabine with immunotherapy for patients with inoperable locally advanced pancreatic cancer. Pancreas.

[28] Kimura Y (2012). Clinical and immunologic evaluation of dendritic cell-based immunotherapy in combination with gemcitabine and/or S-1 in patients with advanced pancreatic carcinoma. Pancreas.

[29] Orbach A (2010). CD40•FasL and CTLA-4•FasL fusion proteins induce apoptosis in malignant cell lines by dual signaling. Am J Pathol.

[30] Galligan L (2005). Chemotherapy and TRAIL-mediated colon cancer cell death: the roles of p53, TRAIL receptors, and c-FLIP. Mol Cancer Ther.

[31] Todaro M (2013). Chemotherapy sensitizes colon cancer initiating cells to Vγ9Vδ2 T cell-mediated cytotoxicity. PLoS One.

[32] Cerwenka A, Lanier LL (2003). NKG2D ligands: unconventional MHC class I-like molecules exploited by viruses and cancer. Tissue Antigens.

[33] Yamamoto K, Fujiyama Y, Andoh A, Bamba T, Okabe H (2001). Oxidative stress increases MICA and MICB gene expression in the human colon carcinoma cell line (CaCo-2). Biochim Biophys Acta.

[34] Miyashita, T. *et al*. Low-dose gemcitabine induces major histocompatibility complex class I-related chain A/B expression and enhances an antitumor innate immune response in pancreatic cancer. *Clin Exp Med* (2015). 10.1007/s10238-015-0394-x 26449615

[35] McGilvray RW (2009). NKG2D ligand expression in human colorectal cancer reveals associations with prognosis and evidence for immunoediting. Clin Cancer Res.

[36] Cho H (2014). MICA/B and ULBP1 NKG2D ligands are independent predictors of good prognosis in cervical cancer. BMC Cancer.

